# Relationship of Perceived Environmental Characteristics to Leisure-Time Physical Activity and Meeting Recommendations for Physical Activity in Texas

**Published:** 2008-12-15

**Authors:** Carole K. Holahan, Katherine S. Velasquez, Xiuhong You

**Affiliations:** The University of Texas at Austin, Department of Kinesiology and Health Education; The University of Texas at Austin, Austin, Texas; The University of Texas at Austin, Austin, Texas

## Abstract

**Introduction:**

We investigated the relationship of perceived environmental characteristics to self-reported physical activity in Texas adults using 2004 Behavioral Risk Factor Surveillance System data.

**Methods:**

The 2 research questions were, "Are perceived neighborhood characteristics and reported use of facilities associated with self-reported leisure-time physical activity for male and female Texas residents aged 18 to 64 years?" and "Are perceived neighborhood characteristics and reported use of facilities related to meeting recommendations for moderate to vigorous physical activity for Texas men and women aged 18 to 64 years?" Descriptive statistics and multiple logistic regression were used for the analyses.

**Results:**

Multiple logistic regression analyses controlling for sociodemographic factors showed that for women, perceptions of neighbors being physically active, pleasantness of the neighborhood, lighting, safety, and feelings of neighbor trustworthiness were associated with leisure-time physical activity. Several of these variables were also related to meeting recommendations for physical activity. Reports of use of several types of neighborhood facilities were related to men's and women's leisure-time physical activity and with meeting recommendations for physical activity for women.

**Conclusion:**

Perceptions of neighborhood characteristics and reported use of facilities were related to physical activity and to meeting recommendations for physical activity, with stronger associations for women than for men. Interventions to increase levels of physical activity among Texans should be informed by multilevel assessments including environmental characteristics and by attention to important subpopulations.

## Introduction

Physical inactivity is a leading preventable cause of death in the United States (1), and increasing physical activity is a leading objective of *Healthy People 2010* (2). The target goal is to reduce leisure-time inactivity from the 1997 baselines of 40% for any race to 20% (3). By 2002, leisure-time inactivity had dropped only to 38%. The 2010 target for engaging in regular moderate or vigorous physical activity is 50% for all races. Yet the participation in regular physical activity in the adult population had increased only from 32% in 1997 to 33% in 2003 (3).

Although many studies have examined the correlates of physical activity in adults, most explored demographic and psychosocial factors. However, a recent trend is to investigate environmental determinants of physical activity (4). Environmental approaches may be particularly useful complements to individual change interventions because they have the potential to benefit all people in the environment (5). Moreover, the analysis of factors that are important for particular subgroups may help promote physical activity among various segments of the population.

The study of environmental factors in physical activity is consistent with an ecological approach to the study of health behavior. Sallis and Owen (6) note that ecological models are multilevel and typically focus on environmental causes and interventions. Ecological approaches recognize that "human-environmental interactions" occur at different levels of aggregation: individuals, families, organizations, communities, and whole populations. Specific ecological models may be needed for specific types of activity because different activities occur in different settings. One setting for physical activity is the neighborhood and elements of the community in which the neighborhood is located.

Leisure-time physical activity (LTPA) has frequently been studied as a means of measuring physical activity. LTPA is usually defined as activities outside a regular job, including exercise, sport participation, hobbies that involve physical activity such as gardening, and walking or running. Measures of LTPA often do not differentiate among types of activity. However, in a study using Behavioral Risk Factor Surveillance System (BRFSS) data from 1987 to 2000, walking was the most reported LTPA for men and women of all ages (7).

Environmental conditions that have been studied in relation to LTPA include qualities of the neighborhood, social and cultural norms, availability and accessibility of recreational resources, and community design. Researchers have found that neighborhood characteristics such as hills, enjoyable scenery, presence of sidewalks and lighting, and safety were associated with more leisure-time activity (4,5,8-13). Several studies found that seeing others walking in the neighborhood was positively correlated with LTPA or walking (4,11,12,14). Numerous studies have found lack of availability and inaccessibility of recreational resources to be deterrents to physical activity (4,11,13,15-17). Accessibility factors include distance, lack of transportation, cost, and number and type of facilities. Finally, higher levels of physical activity have been found in more walkable communities (18-20); in communities with access to attractive, large open spaces (13,21); and in communities with desirable destinations, such as parks, trails, and stores (16,22).

Sex differences in physical activity participation have been identified, with women generally being more inactive. In 1994, 27.9% of men compared with 31.5% of women were inactive, whereas in 2004, 21.4% of men compared with 25.9% of women engaged in no LTPA (23).

Social environmental factors related to physical activity may differ by sex. Some studies have found stronger associations between neighbors walking and physical activity for women than for men (5,24), and several studies with female participants found a positive association of perceived physical activity of neighbors who participated in physical activity (4,11,12,25-27). Perceived safety is frequently studied in scales of varying items that might include traffic, lighting, and crime. Although most studies have failed to find a relationship between safety and physical activity (4,5,11,24-30), a few studies have reported positive results, particularly for women (8,10,12).

The importance of perceived aesthetics and other characteristics of the physical environment may also differ for women and men (24,31,32). Pleasantness has been considered to be enjoyable scenery or aesthetics and to be a composite variable or component of variables, with mixed findings (5,8-10,14,22,29,30,32,33). However, in studies examining women only, associations were generally positive and significant (4,11,12).

The associations between lighting and sidewalks and physical activity have also been studied, although no consistent pattern by sex has emerged. Although 1 study found a significant association between lighting and physical activity for women (8), several other studies (4,5,9,11,22,25-28) showed nonsignificant results. The perceived presence of sidewalks was related to physical activity for both men and women in 2 studies (5,22) but was not related to physical activity in others (4,8,9,11,15,24-28,30).

Although positive relations have been found between perceptions of accessibility and availability of facilities, the findings are mixed. No relation with meeting recommendations was found for shopping malls (5) or for free or low-cost recreation centers (24). Use of or accessibility to neighborhood walking trails, parks, or playgrounds was significantly associated with physical activity variables in several studies (5,18,28,32,33), although findings were nonsignificant in others (15,29). No study we reviewed examined all of these variables concurrently; most studies combined some of them into an "accessibility" factor or as "places to exercise," with mixed findings (8,11,22,24,25,27,28,30).

Trend data from the BRFSS Web site show that Texas leisure-time inactivity data tracked closely with national median data from 1990 until 1998, when they began to diverge, and higher rates of inactivity were seen in Texas (34). Leisure-time physical inactivity was 27.7% nationally and 27.9% in Texas in 1998, whereas in 2002, the inactivity rate was 24.4% nationally and 29.3% in Texas (34). In Texas, the 2004 rates were 23.3% for men and 28.9% for women (overall, 26.1%) (35). The most recent chronic disease indicators related to physical inactivity in Texas have moved in an undesirable direction, with higher overweight and obesity and lower physical activity in adults and youth and higher television viewing by youth (36). In addition, when chronic disease rates are adjusted for age, Texas rates are higher than national rates for cardiovascular disease and diabetes incidence and mortality.

Although environmental and policy strategies are being promoted widely, with the exception of 1 recent study (9), data on the link between environmental factors and physical activity in Texas populations are sparse. We investigated perceived environmental characteristics as correlates of self-reported physical activity in Texas adults by using 2004 BRFSS data. The 2 research questions addressed by this study are, "Are perceived neighborhood characteristics and reported use of facilities associated with self-reported leisure time physical activity for male and female Texas residents aged 18 to 64 years?" and "Are perceived neighborhood characteristics and reported use of facilities related to meeting recommendations for moderate to vigorous physical activity for Texas men and women aged 18 to 64 years?"

## Methods

We analyzed 2004 Texas BRFSS data. The BRFSS is a state-based telephone survey that gathers data on risk factors among noninstitutionalized adults aged 18 years or older. Random-digit dialing and multistage cluster sampling are used to select participants and ensure that a representative sample is drawn. Personnel trained in computer-assisted telephone interviewing techniques administer the questionnaire, which is available in both English and Spanish. Once data collection is completed, all states submit data to the Centers for Disease Control and Prevention where it is aggregated and assigned sample weights. These procedures create estimates that are representative of each state's population and account for sampling error. The sample for this study included adults aged 18 through 64 years who were residing in Texas (N = 6,317).

Information gathered through the BRFSS is used to track indicators that are linked to chronic disease, illness, and health promotion. States typically use BRFSS data to set priorities and determine health policy objectives. The BRFSS questionnaire contains 3 types of questions: core, optional, and state-added. Core questions, which must be administered annually and without modification by all states, include demographic characteristics and health behavior. Optional module questions focus on specific topic areas. Questions that address local issues and priorities are state-added questions. This study used core questions that addressed demographic characteristics (age, sex, education, race/ethnicity, and income), LTPA, and state-added questions regarding moderate and vigorous physical activity and neighborhood characteristics.

### Measures

**Demographic variables**

Demographic variables used in our study included age, sex, education (less than high school graduate, high school graduate or some college, and college graduate), annual income (<$25,000, $25,000 to <$75,000, and $75,000 or more), and race/ethnicity (white, black, Hispanic, and other).

**Physical activity**

The BRFSS core defines LTPA as a "yes" response to the question, "During the past month, other than your regular job, did you participate in any physical activities or exercise, such as running, calisthenics, golf, gardening, or walking for exercise?" The state also added 7 questions regarding moderate and vigorous activity. Interviewers read the following description of moderate and vigorous activity: "Vigorous activities cause large increases in breathing or heart rate while moderate activities cause small increases in breathing or heart rate" (36). Questions determined days per week and minutes per day spent doing moderate and vigorous activities. The state health department transformed data from these questions into a trichotomized variable indicating whether individuals met recommended amounts of physical activity per week, did not meet recommended amounts but were physically active, or were not physically active at all. Individuals met the recommendation if they engaged in 30 minutes of moderate activity 5 days per week or 20 minutes of vigorous activity 3 days per week. The trichotomized variable was used for analysis in this study.

**Neighborhood characteristics**

The neighborhood characteristic variables included 6 state-added questions regarding neighborhood. Respondents were asked to characterize the people, in general, in their neighborhood with 4 response options ranging from "not at all physically active" to "very physically active." Respondents rated their neighborhood as a place to walk with 4 response options ranging from "not pleasant at all" to "very pleasant." They were asked to describe the street lighting in their neighborhood for walking at night with 5 response options ranging from "very poor" to "very good." Respondents were asked to describe how safe from crime they consider their neighborhood with 4 response options ranging from "not at all safe" to "extremely safe." They were also asked if most people in their neighborhood can be trusted (yes/no), and if their neighborhood has any sidewalks (yes/no).

**Neighborhood facilities**

Respondents were asked about their use of several facilities in their community for physical activity. These included public recreation centers, schools that are open for public recreation, private or membership-only recreation facilities, walking trails, parks, playgrounds or sports fields, and shopping malls. Respondents indicated whether they used the facilities (yes/no) or that the community does not have that type of facility.

### Analysis

To accommodate the complex sampling design of the BRFSS, SPSS version 13.0 (SPSS Inc, Chicago, Illinois) was used to perform statistical analyses. Descriptive statistics were computed for each of the major variables. The models were run separately by sex. Each variable was analyzed separately by sex in 2 sets of logistic regressions adjusted for demographic variables — 1 set to explore LTPA and 1 set to examine meeting recommendations for moderate to vigorous physical activity. The state-added questions on neighborhood characteristics and facilities were the independent variables, and each of the 11 neighborhood variables was included as an independent variable. Each neighborhood variable was tested while controlling for education, income, race/ethnicity, and age. These covariates consistently appear as significant correlates of physical activity in national studies (5). Statistical significance for all analyses was set at α = .05. Analyses conducted for this study were approved by the University of Texas institutional review board.

## Results

The sample was 50.7% female, 51% had graduated from high school and may have had some college, 42.5% earned $25,000 to less than $75,000 annually, 59% were white, and 29.5% were Hispanic (Table 1). Of the sample, 74% participated in LTPA, and 26% were physically inactive outside of regular work. Using the state algorithm for meeting recommended physical activity, 44.2% met recommendations, 40.5% engaged in some physical activity but did not meet recommendations, and 15.4% engaged in no physical activity.

Table 2 presents logistic regression model results examining the likelihood of participating in LTPA, and Table 3 presents logistic regression model results for meeting recommendations for physical activity. The findings are based on results adjusted for sociodemographic factors (education, age, income, and race/ethnicity).

The perception that people in the neighborhood were somewhat physically active was associated with LTPA for women. In addition, women's perceptions that the neighborhood was very pleasant or somewhat pleasant were associated with LTPA. Perceived neighborhood lighting also related to women's LTPA when levels of lighting were compared with "very poor lighting"; however, these results were significant only for comparisons with "very good" and "poor" lighting. Women who considered their neighborhood extremely safe or quite safe and women who thought that most people in the neighborhood could be trusted also reported LTPA. Men who reported sidewalks in the neighborhood were more likely than women to report engaging in LTPA. For women, perceptions of physical activity levels of neighbors, pleasantness of the neighborhood, and neighborhood safety and feeling that neighbors were trustworthy were all associated with meeting recommendations for physical activity. For men, none of these variables was significantly associated with meeting recommendations.

Reported use of private or membership-only recreation facilities, use of walking trails, parks, playgrounds, or sports fields, and use of public recreation centers were related to LTPA for both men and women; the use of schools open for public recreation was also associated for men. The reported use of walking trails, parks, playground, or sports fields, use of public recreation centers, and use of schools were associated with meeting recommendations for physical activity among women, but none of the facilities items was significant for meeting recommendations among men. The reported use of shopping malls was not significantly related to LTPA or meeting recommendations.

## Discussion

Most perceived neighborhood characteristics and types of neighborhood facilities reported as being used were related to LTPA or meeting recommendations for moderate to vigorous physical activity. However, findings were stronger for women than for men, especially with respect to neighborhood characteristics.

The findings of our study differed in several respects from those of a similar study that used data collected in Austin/Travis County, Texas, using BRFSS methods and the same 6 neighborhood characteristic variables and year (2004) as our study (9). After adjusting for age, sex, race/ethnicity, and education, the researchers found significant associations between neighborhood characteristics and physical activity. People who reported that their neighborhood is extremely safe were more likely to report LTPA. In addition, perceptions of the neighborhood as very pleasant were associated with physical activity. In our study, safety and neighborhood pleasantness were significant for women alone, and the women who rated the neighborhood as quite safe were as likely as those who rated it extremely safe to engage in LTPA compared with those who rated their neighborhood as slightly safe or not at all safe. The analyses of the Austin data did not show associations between LTPA and perceptions of sidewalks, street lighting, or levels of physical activity or trustworthiness of neighbors. In our study, increased likelihood of reporting LTPA was associated, among men, with the reported presence of sidewalks and among women, with the reported presence of street lighting and the perceived physical activity levels and trustworthiness of neighbors.

Perceptions of several social factors showed positive results in our study. Perceptions that people in the neighborhood are "somewhat physically active" were associated with increased LTPA and engaging in some physical activity for women. Additionally, women were more likely to meet recommendations when they perceived people to be physically active at any level as opposed to not at all physically active. These findings are generally consistent with those of previous studies (4,5,11,12,24-27). Although the findings of previous studies are mixed (4,5,8,10-12,24-30), there is also evidence in our study of the relation of perceived safety from crime to physical activity and to meeting recommendations for women. Safety was also significant in the Austin/Travis County, Texas, study (9). Finally, neighbor trustworthiness was positively associated with LTPA and meeting recommendations for women in our study, although it was nonsignificant (9) or was not addressed in other studies.

Among perceptions of the physical characteristics of neighborhoods, perceived pleasantness of the neighborhood was associated with both LTPA and meeting recommendations for women in our study, although findings of other studies have been mixed (4,5,8-12,15,22,29,30,32,33). Even "not very pleasant" compared with "not pleasant at all" was significantly associated with meeting recommendations for women.

Our study indicated associations between perceptions of lighting and sidewalks and physical activity, in agreement with a few previous studies. Perceived lighting was significantly associated with increased LTPA for women in our study, consistent with findings of Suminski et al (8). Perceived presence of sidewalks was significantly associated with increased LTPA for men in our study, and for both men and women in other studies (5,22).

Perceived accessibility and availability of places to be physically active in the neighborhood, which have shown mixed findings in relation to physical activity in other studies (5,8,11,15,18,22,24,25,27-30,32,33), showed more consistent relations in our study. In this study, reported use of all of the neighborhood facilities variables with the exception of shopping malls showed some significant relation with LTPA and/or meeting recommendations for physical activity. Increased LTPA in both sexes was associated with use of private recreation facilities; use of walking trails, parks, playgrounds, or sports fields; and use of public recreation centers. In addition, use of schools open for public recreation was associated with increased LTPA in men. Several of these variables were also related to meeting recommendations for women. Lack of consistency in the findings for accessibility and availability of places to exercise in other studies may be attributed to differences in item wording or components in scales and to methods for determining the presence of facilities.

Clearly, the findings regarding environmental characteristics are inconsistent, which may be the result of several factors, including differences by sex. First, measurement issues, such as labeling, scale construction, self-report versus objective measurement, sample size, dependent variable definitions, and physical activity behavior (ie, walking vs other activity) make cross-study comparisons difficult and findings inconsistent. Of particular interest is the categorization of active status. Some studies dichotomize by active or not active, whereas other studies dichotomize the dependent variable into meeting recommendations or insufficient and no activity. Our study trichotomized physical activity into meeting recommendations, some activity, and no activity. For selected independent variables in our study, we found differences in relationships at all 3 levels of the dependent variable, demonstrating a potential loss of information for studies in which the variable is dichotomized. Second, some interaction or moderating effects may not have been considered. For example, people in unsafe or unpleasant neighborhoods could be choosing to exercise away from the neighborhood or in their homes. The differences between men and women in our study demonstrate a moderating effect of sex that needs to be examined more closely.

The strong relation of perceived neighborhood characteristics in our study to physical activity in women may be related to mode of exercise and where activity occurs. Women appear more likely than men to engage in walking for physical activity (8,38,39) and to engage in activity in the neighborhood (8). Women may prefer walking in their own neighborhood as their physical activity. Moreover, preference for walking may be related to the findings that multiple role demands with families and lack of time are particular barriers to their participation in physical activity (31,40). Plainly, the findings of our study and those of others demonstrate the need for population- and behavior-specific ecological models (6).

BRFSS data are self-reported and have not been independently verified. People may use different standards in assessing environmental quality, and they may differ in their knowledge of the environment. Moreover, our study was cross-sectional in nature and cannot be interpreted as demonstrating causality. It is possible, for example, that people who are physically active may have chosen to move to a particular neighborhood because of opportunities for physical activity.

This study is subject to several additional limitations. The BRFSS is a telephone survey that is open to nonresponse and selection bias. All information is self-reported, susceptible to recall and social desirability bias, and is not validated by other means. Moreover, the BRFSS may not adequately determine the extent and patterns of physical activity of ethnic minorities because of a narrow definition of physical activity, and may use terms that are unfamiliar to or misunderstood by minority subgroups (4). Finally, most respondents to the 2004 BRFSS came from metropolitan statistical areas and, therefore, may not fully represent the population of Texas.

Despite limitations of the BRFSS and methodologic challenges of studying environmental correlates of physical activity, exploration of environmental factors using BRFSS can produce information to guide interventions for targeted populations in Texas. Qualities of specific neighborhoods and communities across Texas urban and rural areas need to be examined. The findings of such studies should affect the establishment of interventions and legislation and policy regarding community design and use of tax dollars for recreational programming and facilities, and for ensuring safety for physical activity. These interventions should be specific to a neighborhood and its residents. The varied factors associated with physical activity and the pattern of results in our study underscore the importance of a multilevel, ecological approach to understanding physical activity and the likelihood that sex is a important moderating factor.

## Figures and Tables

**Table 1:** Participant Characteristics and Responses to Physical Activity-Related Questions, Behavioral Risk Factor Surveillance System, Texas, 2004^a^

**Variable**	**Total Sample (n = 6,317), % (SE)**	**Men (n = 2,372), %**	**Women (n = 3,945), %**
**Mean age, y**	43.8 (0.3)	42.3	43.5
**Sex**			
Male	49.3 (0.8)	NA	NA
Female	50.7 (0.8)	NA	NA
**Education**			
Less than high school graduate	19.4 (0.7)	15.9	16.6
High school graduate or some college	51.0 (0.8)	50.0	55.0
College graduate	29.6 (0.7)	34.0	28.4
**Annual income**			
<$25,000	35.3 (0.8)	30.5	38.0
$25,000 to <$75,000	42.5 (0.8)	44.5	42.0
≥$75,000	22.2 (0.7)	25.5	19.9
**Race/ethnicity**			
White	59.0 (NA)	59.2	58.7
Black	8.0 (NA)	7.8	8.5
Hispanic	29.5 (NA)	29.0	30.0
Other	3.4 (NA)	4.0	2.8
**During the past month, other than your regular job, did you participate in any physical activities or exercise, such as running, calisthenics, golf, gardening, or walking for exercise?**			
Yes	74.0 (0.7)	79.2	72.8
No	26.0 (0.7)	20.8	27.2
**Status on meeting recommendations for moderate or vigorous physical activity^b^**			
Meets recommendations	44.2 (0.8)	49	42.1
Some physical activity but does not meet recommendations	40.5 (0.8)	39.4	41.9
No physical activity	15.4 (0.6)	11.6	16.0
**In general, would you say that the people in your neighborhood are . . .**			
Very physically active	14.1 (0.6)	12.8	15.4
Somewhat physically active	56.9 (0.9)	59.2	54.7
Not very physically active	21.4 (0.8)	20.8	22.0
Not at all physically active	7.5 (0.5)	7.2	7.9
**Overall, how would you rate your neighborhood as a place to walk?**			
Very pleasant	54.4 (0.9)	55.6	53.2
Somewhat pleasant	36.3 (0.8)	36.3	36.2
Not very pleasant	5.6 (0.4)	5.0	6.3
Not at all pleasant	3.7 (0.3)	3.0	4.3
**For walking at night, would you describe the street lighting in your neighborhood as . . .**			
Very good	11.3 (0.5)	11.0	11.6
Good	28.5 (0.8)	29.2	27.9
Fair	27.8 (0.8)	27.9	27.7
Poor	17.8 (0.7)	18.3	17.4
Very poor	14.6 (0.6)	13.7	15.4
**How safe from crime do you consider your neighborhood to be?**			
Extremely safe	18.1 (0.6)	20.1	16.3
Quite safe	50.4 (0.9)	49.4	51.4
Slightly safe	24.0 (0.8)	22.9	25.0
Not at all safe	7.5 (0.5)	7.6	7.3
**Generally speaking, would you say most people in your neighborhood can be trusted?**			
Yes	83.3 (0.7)	84.7	82.0
No	16.7 (0.7)	15.3	18.0
**Does your neighborhood have any sidewalks?**			
Yes	57.1 (0.8)	57.0	57.3
No	42.9 (0.8)	43.0	42.7
**Do you use any private or membership-only recreation facilities in your community for physical activity?**			
Yes	24.4 (0.7)	26.2	22.5
No	71.9 (0.8)	70.1	73.8
My community does not have these facilities	3.7 (0.3)	3.7	3.7
**Do you use walking trails, parks, playgrounds, or sports fields in your community for physical activity?**			
Yes	49.1 (0.9)	51.6	46.5
No	48.6 (0.9)	46.3	51
My community does not have these facilities	2.3 (0.2)	2.1	2.5
**Do you use shopping malls in your community for physical activity and/or walking programs?**			
Yes	17.0 (0.7)	15.5	18.5
No	78.8 (0.7)	81.2	76.5
My community does not have shopping malls	4.1 (0.3)	3.3	5.0
**Do you use any public recreation centers in your community for physical activity?**			
Yes	25.4 (0.8)	29.2	21.8
No	72.2 (0.8)	68.7	75.6
My community does not have these facilities	2.3 (0.3)	2.1	2.6
**Do you use schools that are open in your community for public recreation activities?**			
Yes	21.0 (0.7)	21.9	19.9
No	77.1 (0.7)	76.1	78.1

Abbreviation: SE, standard error; NA, not available.

a Frequencies determined using tde Centers for Disease Control and Prevention's complex sampling design.

b Interviewers read tde following description of moderate and vigorous activity: "Vigorous activities cause large increases in breathing or heart rate while moderate activities cause small increases in breathing or heart rate" (37). Questions determined days per week and minutes per day spent doing moderate and vigorous activities. The state health department transformed data from these questions into a trichotomized variable indicating whether individuals met recommended amounts of physical activity per week, did not meet recommended amounts but were physically active, or were not physically active at all. Individuals met the recommendation if they engaged in 30 minutes of moderate activity 5 days per week or 20 minutes of vigorous activity 3 days per week. The trichotomized variable was used for analysis in this study.

**Table 2:** Odds Ratios^a^ for Texas Adults Who Participated in Leisure-Time Physical Activity, by Sex, Behavioral Risk Factor Surveillance System, Texas, 2004^b^

Question/Response	Men (n = 2,372)		Women (n = 3,945)	

	Odds Ratio (95% CI)	*P* value	Odds Ratio (95% CI)	*P* value
**In general, would you say that the people in your neighborhood are . . .**				
Very physically active	0.92 (0.48-1.78)	.81	1.43 (0.86-2.37)	.17
Somewhat physically active	1.39 (0.80-2.44)	.25	1.62 (1.05-2.51)	.03
Not very physically active	0.93 (0.49-1.74)	.82	1.18 (0.74-1.88)	.49
Not at all physically active	1 [Reference]		1 [Reference]	
**Overall, how would you rate your neighborhood as a place to walk?**				
Very pleasant	1.76 (0.64-4.86)	.27	2.22 (1.38-3.55)	.001
Somewhat pleasant	1.44 (0.52-3.97)	.48	1.81 (1.13-2.90)	.01
Not very pleasant	1.62 (0.53-5.00)	.40	1.42 (0.80-2.53)	.23
Not pleasant at all	1 [Reference]		1 [Reference]	
**For walking at night would you describe the street lighting in your neighborhood as . . .**				
Very good	0.85 (0.49-1.47)	.55	1.70 (1.14-2.54)	.01
Good	1.20 (0.75-1.93)	.45	1.31 (0.95-1.81)	.10
Fair	1.11 (0.70-1.75)	.66	1.31 (0.94-1.83)	.11
Poor	1.03 (0.61-1.74)	.90	1.44 (1.02-2.04)	.04
Very poor	1 [Reference]		1 [Reference]	
**How safe from crime do you consider your neighborhood to be?**				
Extremely safe	1.05 (0.51-2.15)	.90	1.70 (1.06-2.73)	.03
Quite safe	1.49 (0.77-2.88)	.24	1.75 (1.16-2.63)	.008
Slightly safe	1.19 (0.61-2.32)	.61	1.52 (0.99-2.32)	.05
Not at all safe	1 [Reference]		1 [Reference]	
**Generally speaking, would you say most people in your neighborhood can be trusted?**				
Yes	1.07 (0.66-1.75)	.78	1.35 (1.00-1.82)	.05
No	1 [Reference]		1 [Reference]	
**Does your neighborhood have any sidewalks?**				
Yes	1.47 (1.09-1.99)	.01	1.03 (0.82-1.28)	.82
No	1 [Reference]		1 [Reference]	
**Do you use any private or membership-only recreation facilities in your community for physical activity?**				
Yes	2.32 (1.05-5.16)	.04	2.30 (1.32-4.02)	.003
No	0.94 (0.46-1.92)	.87	0.79 (0.49-1.29)	.35
My community does not have these facilities	1 [Reference]		1 [Reference]	
**Do you use walking trails, parks, playgrounds, or sports fields in your community for physical activity?**				
Yes	2.45 (1.03-5.83)	.04	2.18 (1.24-3.83)	.007
No	0.89 (0.38-2.06)	.79	0.75 (0.43-1.29)	.30
My community does not have these facilities	1 [Reference]		1 [Reference]	
**Do you use shopping malls in your community for physical activity and/or walking programs?**				
Yes	1.39 (0.61-3.19)	.44	0.96 (0.59-1.56)	.87
No	0.97 (0.46-2.03)	.93	0.63 (0.41-0.97)	.04
My community does not have shopping malls	1 [Reference]		1 [Reference]	
**Do you use any public recreation centers in your community for physical activity?**				
Yes	5.08 (2.08-12.41)	<.001	2.26 (1.23-4.15)	.009
No	2.07 (0.90-4.79)	.09	1.05 (0.61-1.83)	.86
My community does not have these facilities	1 [Reference]		1 [Reference]	
**Do you use schools that are open in your community for public recreation activities?**				
Yes	3.83 (1.46-10.04)	.006	1.80 (0.91-3.56)	.09
No	1.77 (0.75-4.17)	.19	1.15 (0.60-2.19)	.67
Schools in my community are not open for public use	1 [Reference]		1 [Reference]	

Abbreviation: CI, confidence interval; Ref, reference.

a Adjusted for age, education, income, and race/ethnicity.

b Separate logistic regression analysis using the Centers for Disease Control and Prevention's complex sampling design was used for each neighborhood variable; therefore, data were weighted to reflect the Texas population. Cases with missing data were not included; therefore, number of respondents varies for some models.

**Table 3:** Odds Ratios^a^ (ORs) for Texas Adults (Aged 18 to 65) Who Met Recommendations for Moderate or Vigorous Physical Activity or Some Activity^b^, by Sex, Behavioral Risk Factor Surveillance System, Texas, 2004^c^

Question/Response	**Men**		**Women**	

	**Meets Recommendations, OR (95% CI)**	**Some Activity, OR (95% CI)**	**Meets Recommendations, OR (95% CI)**	**Some Activity, OR (95% CI)**
**In general, would you say that the people in your neighborhood are. . .**				
Very physically active	1.76 (0.76-4.10)	1.50 (0.62-3.61)	2.26 (1.16-4.43)	1.33 (0.70-2.53)
Somewhat physically active	1.81 (0.86-3.80)	1.69 (0.79-3.64)	2.58 (1.42-4.68)	2.20 (1.27-3.80)
Not very physically active	1.12 (0.50-2.52)	1.39 (0.61-3.18)	2.20 (1.17-4.14)	1.78 (0.99-3.18)
Not at all physically active	1 [Reference]	1 [Reference]	1 [Reference]	1 [Reference]
**Overall, how would rate your neighborhood as a place to walk?**				
Very pleasant	1.27 (0.47-3.44)	2.73 (0.91-8.21)	3.79 (1.93-7.45)	1.93 (1.04-3.58)
Somewhat pleasant	0.61 (0.22-1.68)	1.50 (0.49-4.58)	3.14 (1.04-3.58)	1.93 (1.03-3.60)
Not very pleasant	0.90 (0.26-3.06)	2.01 (0.53-7.59)	2.55 (1.58-6.23)	1.74 (0.81-3.76)
Not pleasant at all	1 [Reference]	1 [Reference]	1 [Reference]	1 [Reference]
**For walking at night would you describe the street lighting in your neighborhood as…**				
Very good	0.96 (0.47-1.96)	0.85 (0.40-1.79)	0.85 (0.49-1.48)	0.80 (0.46-1.39)
Good	0.75 (0.41-1.36)	0.82 (0.44-1.53)	0.83 (0.52-1.34)	1.11 (0.70-1.78)
Fair	1.12 (0.59-2.11)	1.33 (0.68-2.57)	1.04 (0.65-1.67)	1.18 (0.74-1.88)
Poor	0.81 (0.41-1.59)	0.90 (0.45-1.83)	1.34 (0.80-2.23)	1.47 (0.88-2.44)
Very poor	1 [Reference]	1 [Reference]	1 [Reference]	1 [Reference]
**How safe from crime do you consider your neighborhood to be?**				
Extremely safe	1.23 (0.53-2.87)	1.49 (0.62-3.57)	2.17 (1.11-4.21)	1.76 (0.91-3.39)
Quite safe	1.48 (0.68-3.57)	2.40 (1.07-5.39)	2.24 (1.28-3.92)	1.81 (1.05-3.12)
Slightly safe	0.80 (0.36-1.76)	1.59 (0.70-3.60)	1.49 (0.84-2.65)	1.54 (0.88-2.70)
Not at all safe	1 [Reference]	1 [Reference]	1 [Reference]	1 [Reference]
**Generally speaking, would you say most people in your neighborhood can be trusted?**				
Yes	1.29 (0.73-2.29)	1.28 (0.71-2.33)	1.61 (1.08-2.41)	1.46 (0.99-2.17)
No	1 [Reference]	1 [Reference]	1 [Reference]	1 [Reference]
**Does your neighborhood have any sidewalks?**				
Yes	0.92 (0.61-1.39)	1.29 (0.85-1.94)	0.90 (0.67-1.22)	0.98 (0.73-1.31)
No	1 [Reference]	1 [Reference]	1 [Reference]	1 [Reference]
**Do you use any private or membership only recreation facilities in your community for physical activity?**				
Yes	1.96 (0.67-5.71)	2.30 (0.76-6.99)	1.79 (0.78-4.10)	1.13 (0.51-2.54)
No	0.57 (0.23-1.42)	1.08 (0.42-2.78)	0.91 (0.44-1.91)	0.84 (0.41-1.71)
My community does not have these facilities	1 [Reference]	1 [Reference]	1 [Reference]	1 [Reference]
**Do you use walking trails, parks, playgrounds, or sports fields in your community for physical activity?**				
Yes	1.62 (0.55-4.81)	2.37 (0.74-7.57)	2.37 (1.09-5.14)	2.91 (1.32-6.45)
No	0.58 (0.20-1.70)	1.12 (0.36-3.48)	0.89 (0.42-1.86)	1.33 (0.62-2.85)
My community does not have these facilities	1 [Reference]	1 [Reference]	1 [Reference]	1 [Reference]
**Do you use shopping malls in your community for physical activity and/or walking programs?**				
Yes	0.76 (0.27-2.15)	1.66 (0.56-4.93)	0.93 (0.49-1.79)	0.92 (0.48-1.76)
No	0.57 (0.23-1.39)	0.89 (0.34-2.30)	0.84 (0.47-1.49)	0.75 (0.42-1.32)
My community does not have shopping malls	1 [Reference]	1 [Reference]	1 [Reference]	1 [Reference]
**Do you use any public recreation centers in your community for physical activity?**				
Yes	1.21 (0.37-3.94)	0.72 (0.20-2.57)	2.87 (1.26-6.54)	1.92 (0.80-4.57)
No	0.76 (0.24-2.37)	0.54 (0.16-1.85)	1.26 (0.59-2.68)	1.01 (0.45-2.24)
My community does not have these facilities	1 [Reference]	1 [Reference]	1 [Reference]	1 [Reference]
**Do you use schools that are open in your community for public recreation activities?**				
Yes	2.23 (0.61-8.21)	1.31 (0.32-5.35)	2.57 (1.02-6.42)	3.61 (1.33-9.82)
No	0.79 (0.24-2.65)	0.59 (0.16-2.18)	1.30 (0.56-3.05)	2.59 (1.01-6.61)
Schools in my community are not open for public use	1 [Reference]	1 [Reference]	1 [Reference]	1 [Reference]

Abbreviation: CI, confidence interval; Ref, reference.

a Adjusted for age, education, income, and race/ethnicity.

b Interviewers read the following description of moderate and vigorous activity: "Vigorous activities cause large increases in breathing or heart rate while moderate activities cause small increases in breathing or heart rate" (37). Questions determined days per week and minutes per day spent doing moderate and vigorous activities. The state health department transformed data from these questions into a trichotomized variable indicating whether individuals met recommended amounts of physical activity per week, did not meet recommended amounts but were physically active, or were not physically active at all. Individuals met the recommendation if they engaged in 30 minutes of moderate activity 5 days per week or 20 minutes of vigorous activity 3 days per week. The trichotomized variable was used for analysis in this study.

c Separate logistic regression analysis using the Centers for Disease Control and Prevention's complex sampling design was used for each neighborhood variable; therefore, data were weighted to reflect the Texas population. Cases with missing data were not included; therefore, number of respondents varies for some models.
